# Announcement: 4th Anglo/German Inorganic Chemistry Meeting (AGICHEM '97)

**DOI:** 10.1155/MBD.1997.53

**Published:** 1997

**Authors:** Ch. Elschenbroich

**Affiliations:** Fachbereich Chemie Philipps-Universität Marburg Hans-Meerwein-Straße Marburg D-35032 Germany


					Gesellschaft Deutscher Chemiker
W6hler-Vereinigung far Anorganische Chemie
an0
Royal Society of Chemistry, Dalton Division
AGICH M '97
4th Anglo/German Inorganic Chemistr Meeting
Invitation and Call for Posters
Marburg, Germany
September 14 -17, 1997
General Information
The series of Anglo/German Inorganic Chemistry Meetings (AGICHEM) was inaugurated in 1991 as a
joint venture of the Gesellschaft Deutscher Chemiker and the Royal Society of Chemistry. The first
meeting at Brighton was chaired by M. Lapped and H. Noth. Subsequent conferences were held at
Stuttgart (1993, A. Simon, M. Lappert) and Brighton (1995, J. Nixon, H. Noth). They have
established themselves as a highly effective forum for the exchange of new concepts and ideas in
inorganic chemistry. This can be traced to manageable size, the absence of parallel sessions and a
cordial atmosphere.
Location and Dates
Marburg was formerly one of the great pilgrimage centers of the West, the crowds being drawn by
the relics of Elizabeth of Hungary. It is the site of the oldest existing protestant university founded in
1527, a magnificient gothic cathedral and old quarters which are interspersed by twisting and
climbing alleys. Marburg is situated 100 km north of Frankfurt am Main and can be reached from
there by an hourly train service.
The meeting starts Sunday 14 September 1997 with an evening reception and ends Wednesday
17 September 1997 around noon.
Accomodation
Accomodation will be available in hotels and in student dormitories all at walking distance from the
conference site. Further details will be provided in the second circular.
53
Scope and Programme
AGICHEM '97 is intended to present new developments from all fields of Inorganic Chemistry.
Therefore, the following topics will be adressed:
Organometallic Chemistry
Bioinorganic/Coordination Chemistry
Main Group Chemistry
Solid State/Structural Chemistry
The programme will comprise 26 invited lectures and a large presentation of posters. The latter form
an indispensable part of the conference and two extended periods will be set a side for viewing and
discussion. All posters should be on display during the whole duration of the conference.
The following scientists have agreed to present lectures:
J. Caro, Berlin-Adlershof
U. Diefenbach, Berlin
M. Driess, Heidelberg
R. Dronskowski, Stuttgart
G. Erker, MOnster
R. Fischer, Heidelberg
K.-D. Franz, Darmstadt
H. Hillebrecht, Freiburg
B. Lippert, Dortmund
J. J. Schneider, Essen
M. Tacke, Karlsruhe
F. Tuczek, Mainz
M. Westerhausen, Stuttgart
F. Armstrong, Oxford
D. Cole-Hamilton, St. Andrews
N. Connelly Bristol
J. Errington Newcastle
B. Haywood, Salford
T. Kee, Leeds
R. Mulvey, Strathclyde
D. O Hare, Oxford
R. Perutz, York
A. Powell, East Anglia
K. Prassides, Sussex
D. Rankin, Edinburgh
Organizing Committee
Philipps-Universit&t at Marburg
Ch. Elschenbmich (Chair)
K. Dehnicke
B. Harbrecht
J. Lorberth
J. Sundermeyer
University of Sussex at Brighton
J. F. Nixon (Co-Chair)
Correspondence
Inquiries concerning scientific matters and local organization should be addressed to
Professor Dr. Ch. Elschenbroich
Fachbereich Chemie, Philipps-Universit&t Marburg, Hans-Meerwein-Strale
D-35032 Marburg, Germany
Phone: + 49 69 6421 28 55 27, 5687, Fax: + 49 69 6421 28 89 17
E-mail: EB@ ps1515.chemie.uni-marburg.de
Replies to the second circular and final registrations should be directed to
Gesellschaft Deutscher Chemiker, Abt. Tagungen
Postfach 90 04 40, D-60444 Frankfurt am Main, Germany
Phone: + 49 69 7917 358/-360/-366, Fax: + 49 69 7917 475
E-mail: tg@gdch.de
GDCh-Homepage: http://www.gdch.de
Anybody interested in receiving the second cimular, in attending the conference and in presenting
a poster is asked to contact
Prof. Dr. Ch. Elschenbroich
Fachbereich Chemie, Philipps-Universitit Marburg
Hans-Meerwein-StraBe, D-35032 Marburg, Germany
54

				

